# Factors and preventive strategies for perioperative euglycemic diabetic ketoacidosis in patients with type 2 diabetes receiving sodium-glucose cotransporter 2 inhibitors: a retrospective study

**DOI:** 10.1186/s40780-025-00487-6

**Published:** 2025-08-25

**Authors:** Miho Takemura, Kenji Ikemura, Masahiro Okuda

**Affiliations:** 1 Department of Clinical Pharmacy Research and Education, Graduate School of Pharmaceutical Sciences, The University of Osaka, 1-6 Yamadaoka, Suita, 565-0871 Osaka Japan; 2 Department of Pharmacy, The University of Osaka Hospital, 2-15 Yamadaoka, Suita, 565-0871 Osaka Japan; 3 Department of Hospital Pharmacy, Graduate School of Medicine, The University of Osaka, 2-15 Yamadaoka, Suita, 565-0871 Osaka Japan

**Keywords:** Sodium-glucose cotransporter 2 inhibitor, Euglycemic diabetic ketoacidosis, Risk factors, Prevention strategies

## Abstract

**Background:**

Invasive treatment and the associated stress are known risk factors for sodium-glucose cotransporter 2 inhibitor (SGLT2i)-induced euglycemic diabetic ketoacidosis (euDKA) development. It is recommended that SGLT2is is discontinued at least 3 days prior to a scheduled surgery. However, it is unclear whether preoperative discontinuation of SGLT2is is effective and whether other factors contribute to the development of SGLT2i-induced perioperative euDKA.

**Methods:**

We retrospectively investigated the incidence of euDKA postoperatively up to 30 days in patients receiving SGLT2is and undergoing surgery under general anesthesia. Multivariate logistic regression analysis was performed to identify the factors affecting euDKA development.

**Results:**

Twenty-one of 1,169 eligible patients (1.8%) developed perioperative euDKA. The incidence of perioperative euDKA in patients who discontinued SGLT2is for ≥ 3 days prior to surgery was significantly lower than that in patients who did not discontinue SGLT2is for ≥ 3 days prior to surgery (*p* < 0.001). The multivariate analysis showed that discontinuation of SGLT2is for ≥ 3 days prior to surgery and preoperative use of insulin and glucose infusion were significant factors that affected the development of perioperative euDKA (odds ratios = 0.047 and 0.054, *p* = 0.003 and 0.005, respectively).

**Conclusions:**

Our findings suggest that preoperative SGLT2i discontinuation for at least 3 days could prevent perioperative euDKA development and that preoperative insulin and glucose infusion could reduce the risk of developing euDKA, even in patients who cannot discontinue SGLT2is at least 3 days preoperatively.

**Supplementary Information:**

The online version contains supplementary material available at 10.1186/s40780-025-00487-6.

## Background

Sodium-glucose cotransporter 2 inhibitors (SGLT2is) target the proximal tubules of the kidney to block glucose reabsorption, thereby enhancing urinary glucose excretion and conferring anti-hyperglycemic effects that are primarily insulin-independent [1]. SGLT2is have pronounced glucose-lowering effects with a low risk of causing hypoglycemia when administered as monotherapy [2, 3]. Moreover, recent large-scale clinical trials have demonstrated that SGLT2is reduce the risk of myocardial infarction, heart failure, renal failure, cardiovascular mortality, and all-cause mortality among patients with type 2 diabetes who had high cardiovascular risk [4, 5, 6, 7]. Thus, the use of SGLT2is has steadily increased in recent years owing to their effective glycemic control and other benefits.

In 2015, the U.S. Food and Drug Administration (FDA) issued a warning regarding the potential for SGLT2is to cause euglycemic diabetic ketoacidosis (euDKA), a life-threatening adverse effect [8]. Numerous studies have documented instances of euDKA, which is generally characterized by increased anion gap metabolic acidosis with ketosis but in the setting of normal blood glucose levels, in patients with type 2 diabetes treated with SGLT2is [9, 10, 11]. A previous study identified invasive treatment and the associated stress as risk factors for SGLT2i-induced euDKA [12]. In response to major surgical stimuli, the physiological release of counter-regulatory hormones such as epinephrine, cortisol, and inflammatory mediators and acute insulin resistance stimulate gluconeogenesis, glycogenolysis, and ketogenesis [13]. Therefore, patients receiving SGLT2is in the perioperative setting are at a particularly high risk of developing euDKA, owing to disrupted glucose homeostatic mechanisms and a further decrease in the insulin-to-glucagon ratio associated with SGLT2i use.

In 2020, the FDA updated the recommendations for SGLT2is, advising the discontinuation of SGLT2is at least 3 days prior to surgery [14]. The Japanese package inserts for SGLT2is also recommend discontinuing SGLT2is at least 3 days prior to surgery. In the surgical setting, the recommendations include (i) comprehensive preoperative assessment, (ii) perioperative discontinuation of SGLT2is for ≥ 3 days prior to surgery, (iii) delaying nonemergent surgery if SGLT2is have not been withheld, and (iv) delayed postoperative restart of SGLT2is until the patient has resumed normal eating. However, it is not clear whether discontinuation of SGLT2is for ≥ 3 days prior to surgery is effective in preventing perioperative euDKA development because only a few studies have validated the updated recommendations regarding the discontinuation of SGLT2is. Moreover, although timely discontinuation of SGLT2is may not be feasible in certain emergency situations, no preventive strategy has been established for high-risk patients who cannot discontinue SGLT2is as scheduled to safely undergo emergency surgery without developing euDKA. The aim of this study was to investigate the effect of discontinuing SGLT2is at least 3 days prior to surgery on perioperative euDKA development and the factors affecting the development of perioperative euDKA in patients receiving SGLT2is.

## Methods

### Patients selection

Data on 1,172 in-patients with type 2 diabetes receiving an SGLT2i (dapagliflozin, empagliflozin, canagliflozin, ipragliflozin, tofogliflozin, or luseogliflozin) and undergoing surgery under general anesthesia at The University of Osaka Hospital between January 2014 and December 2023 were extracted from electronic medical records. As it has been reported that an SGLT2i prescription duration exceeding 7 days significantly increased the incidence of postoperative euDKA [15], two patients who initiated SGLT2is within 7 days prior to surgery and one patient who had developed euDKA prior to surgery were excluded.

During preoperative consultation, healthcare providers conducted comprehensive medication reconciliation and provided patients with written instructions on adjusting their type 2 diabetes medications, including perioperative discontinuation of SGLT2is, prior to surgery. However, some patients were unable to discontinue SGLT2is for at least 3 days prior to surgery because of emergency surgery or nonadherence to medical instructions.

Insulin and glucose therapy were administered to prevent perioperative euDKA development in patients who were assumed to have insulin deficiency associated with SGLT2i therapy based on the decision of the physicians. Insulin and glucose infusion were started on the day they were admitted to the hospital in most cases and the majority of patients were admitted to the hospital 1 day prior to surgery. In the preoperative and perioperative settings, patients receiving insulin and glucose therapy were continuously administered 4–10 units of insulin with an intravenous infusion of a glucose-containing solution (20–50 g glucose) following endocrinologist consultation. Following the intensive care unit protocol, the blood glucose level was adjusted to be within the target range of 140–180 mg/dL [16]. The insulin and glucose infusion were discontinued when the patients were able to eat and drink after surgery.

### Assessment

In all patients, laboratory data were evaluated and chart documentation was comprehensively reviewed to diagnose euDKA. EuDKA was defined as arterial pH < 7.3 and blood or urine ketone positivity within 30 days postoperatively in patients receiving SGLT2is [17]. The predominant symptoms of euDKA including nausea, vomiting, tachycardia, and abdominal pain [18] were also investigated. Data were collected from the patient medical records and postoperative laboratory parameter were evaluated based on the worst values within 30 days after surgery. The primary endpoint was the incidence of euDKA within the first 30 days after surgery. The secondary endpoints were factors influencing perioperative euDKA development and their odds ratios (ORs).

### Statistical analyses

Statistical comparisons between patients who discontinued and those who did not discontinue SGLT2is for ≥ 3 days prior to surgery were performed using the Mann–Whitney U-test and Fisher’s exact test for continuous and categorical variables, respectively. Statistical comparison among multiple groups were performed using the Kruskal–Wallis test and a Chi-square test of independence for continuous and categorical variables, respectively. Multivariate logistic regression analysis was performed to identify the factors affecting euDKA development within the first 30 days after surgery. Based on previous studies reporting that age, gender, and body mass index (BMI) are risk factors for perioperative euDKA [17, 19], the logistic regression model was adjusted for the following potential confounding factors: age, sex, BMI, discontinuation of SGLT2is for ≥ 3 days prior to surgery, and preoperative use of insulin and glucose infusion. The statistical analyses were performed using JMP^®^ Pro version 14.3.0 (SAS Institute, Cary, NC, USA). Statistical significance was set at a two-tailed *p*-value < 0.05 and the confidence interval was set to 95%.

## Results

### Background information of patients

After considering the inclusion and exclusion criteria, 1,169 of the 1,172 patients were enrolled in the present study. Six hundred and twenty-seven patients (53.6%) discontinued SGLT2is for ≥ 3 days prior to surgery. Among the patients who did not discontinue SGLT2is for ≥ 3 days prior to surgery (*n* = 542), SGLT2i use was discontinued 2 days and 1 day prior to surgery in 26 (2.2%) and 49 patients (4.2%), respectively, and SGLT2is were administered on the day of surgery in 467 patients (39.9%). The characteristics of patients who discontinued and those who did not discontinue SGLT2is for ≥ 3 days prior to surgery are summarized in Table 1. No significant differences were observed in the demographic characteristics, including age, sex, BMI, SGLT2i type, and preoperative laboratory profile, between the groups. The use of preoperative insulin and glucose infusion was significantly higher in patients who discontinued SGLT2is for ≥ 3 days prior to surgery than in patients who did not discontinue SGLT2is for ≥ 3 days prior to surgery (*p* = 0.005). In contrast, there was no significant difference in the perioperative outcomes between patients who discontinued and who did not discontinue SGLT2is for ≥ 3 days prior to surgery (Supplementary Table 1).

**Table 1:** Characteristics of the patients

	Discontinuation of SGLT2is for ≥ 3 days prior to surgery		*p*-value
	(+) (*n* = 627)	(−) (*n* = 542)	
Age (years)	69 [26–90]	66 [27–97]	0.251
Male	397 (63.3)	337 (62.2)	0.733
BMI (kg/m^2^)	24.2 [13.1–39.2]	23.8 [14.4–39.6]	0.227
SGLT2i type			
Dapagliflozin	182 (29.0)	164 (30.3)	0.514
Empagliflozin	171 (27.3)	153 (28.2)	
Canagliflozin	118 (18.8)	90 (16.6)	
Ipragliflozin	73 (11.6)	73 (13.5)	
Tofogliflozin	46 (7.3)	41 (7.6)	
Luseogliflozin	37 (5.9)	21 (3.9)	
Other diabetes medications			
None	557 (88.8)	482 (88.9)	0.832
OHA	59 (9.4)	51 (9.4)	
Insulin	10 (1.6)	9 (1.7)	
Insulin and OHA	1 (0.2)	0 (0.0)	
Baseline laboratory parameters			
AST level (U/L)	21 [8–74]	21 [7–74]	0.268
ALT level (U/L)	16 [3–74]	16 [3–74]	0.112
Scr level (mg/dL)	0.84 [0.28–6.88]	0.82 [0.36–6.89]	0.362
BUN level (mg/dL)	17 [5–91]	18 [6–82]	0.213
Serum sodium level (mEq/L)	140 [118–152]	140 [120–151]	0.624
Serum potassium level (mEq/L)	4.2 [3.0–6.0]	4.2 [3.2–6.0]	0.448
Serum chloride level (mEq/L)	106 [92–119]	105 [89–118]	0.156
Arterial pH	7.43 [7.34–7.83]	7.44 [7.33–7.67]	0.950
Serum bicarbonate level (mEq/L)	24.0 [17.0–31.8]	24.3 [15.9–31.5]	0.190
Serum BHBA level (µmol/L)	38 [15–178]	35 [14–174]	0.291
Blood glucose level (mg/dL)	122 [66–295]	123 [63–292]	0.434
Preoperative medication			
Insulin and glucose infusion	342 (54.5)	250 (46.1)	0.005

Values are presented as median [range] or number (%). The data were analyzed using the Mann–Whitney U test or Fisher’s exact test. ALT, alanine transaminase; AST, aspartate transaminase; BHBA, β-hydroxybutyrate; BMI, body mass index; BUN, blood urea nitrogen; OHA, oral hypoglycemic agent; Scr, serum creatinine; SGLT2i, sodium-glucose cotransporter 2 inhibitor

### Incidence of euDKA within the first 30 days after surgery

Table 2 presents the postoperative outcomes. Twenty-one patients (1.8%) developed euDKA within the first 30 days after surgery. Among the 21 patients who developed perioperative euDKA, euDKA was diagnosed within 3 days postoperatively in 9 patients, within 7 days in 17 patients, and within 14 days in 20 patients. The incidence of perioperative euDKA in patients who discontinued SGLT2is for ≥ 3 days prior to surgery was significantly lower than that in patients who did not discontinue SGLT2is for ≥ 3 days prior to surgery (*p* < 0.001). The median days [range] on which postoperative laboratory parameters and blood glucose levels were measured after surgery was 5 [2–25] and 4 [2–26] days, respectively. Although euDKA was diagnosed based on laboratory data in only 21 patients, nausea, vomiting, tachycardia, and abdominal pain were recorded in 222, 78, 6, and 2 patients, respectively. No significant differences in the incidence of nausea, vomiting, tachycardia, and abdominal pain were observed between the groups (*p* = 0.136, 0.725, 0.102, and 0.215, respectively).

**Table 2:** Incidence of euDKA within the first 30 days after surgery

	Discontinuation of SGLT2is for ≥ 3 days prior to surgery		*p*-value
	(+) (*n* = 627)	(−) (*n* = 542)	
Development of perioperative euDKA	1 (0.2)	20 (3.7)	< 0.001
Time to euDKA diagnosis			
Within 3 days	0 (0.0)	9 (1.7)	< 0.001
Within 7 days	1 (0.2)	16 (3.0)	
Within 14 days	1 (0.2)	19 (3.5)	
Within 30 days	1 (0.2)	20 (3.7)	
Predominant symptoms of euDKA			
Nausea	109 (17.4)	113 (20.8)	0.136
Vomiting	40 (6.4)	38 (7.0)	0.725
Tachycardia	1 (0.2)	5 (0.9)	0.102
Abdominal pain	0 (0.0)	2 (0.4)	0.215
Postoperative laboratory parameters			
Arterial pH	7.44 [7.32–7.76]	7.44 [7.27–7.55]	0.580
Serum bicarbonate level (mEq/L)	25.0 [17.0–36.6]	24.3 [11.7–33.1]	0.013
Serum BHBA level (µmol/L)	35 [11–203]	37 [12–574]	0.629
Blood glucose level (mg/dL)	112 [68–257]	112 [70–282]	0.327
Level of postoperative blood glucose			
Hyperglycemia (≥ 125 mg/dL)	167 (26.6)	147 (27.1)	0.895
Mild hyperglycemia (125–250 mg/dL)	158 (25.2)	135 (24.9)	0.946
Hypoglycemia (≤ 60 mg/dL)	0 (0.0)	0 (0.0)	N.A.
Days until eating and SGLT2i are resumed after surgery	1 [0–23]	1 [0–24]	0.614

Values are presented as median [range] or number (%). The data were analyzed using the Mann–Whitney U test or Fisher’s exact test. BHBA, β-hydroxybutyrate; euDKA, euglycemic diabetic ketoacidosis; N.A., not available; SGLT2i, sodium-glucose cotransporter 2 inhibitor

### Factors affecting the development of perioperative euDKA

**Fig. 1:**
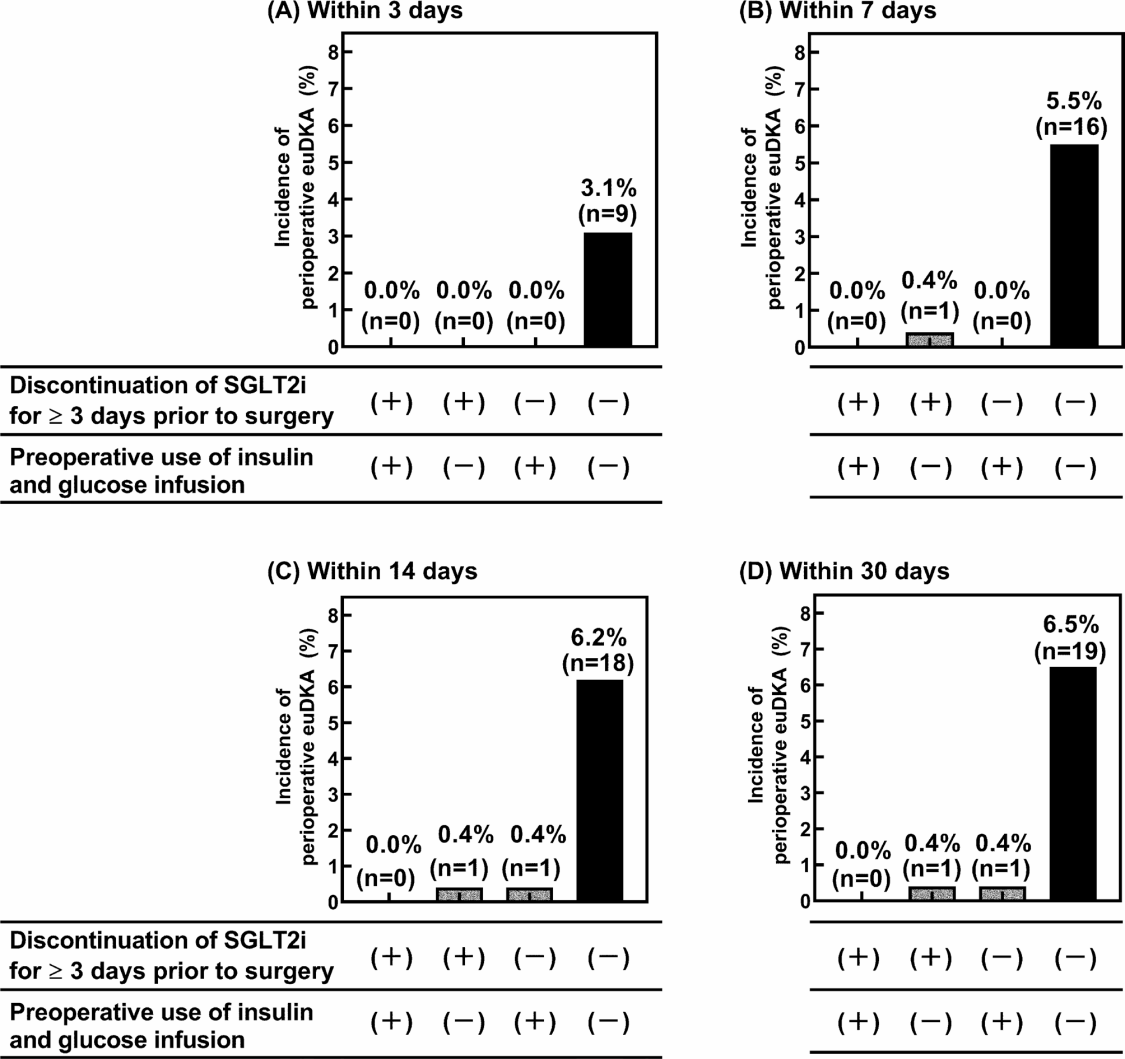
Incidence rate of perioperative euDKA within the first (a) 3 days, (b) 7 days, (c) 14 days, and (d) 30 days after surgery in the four groups of patients: patients who discontinued and those who did not discontinue SGLT2is for ≥ 3 days prior to surgery and patients who received and those who did not receive insulin and glucose infusion preoperatively

A multivariate logistic regression analysis was performed to identify the factors affecting euDKA development within the first 30 days after surgery (Table 3). Among the potential clinical prognostic factors, the multivariate analysis revealed the discontinuation of SGLT2is for ≥ 3 days prior to surgery and preoperative use of insulin and glucose infusion as significant factors that affected the development of perioperative euDKA (ORs = 0.047 and 0.054, *p* = 0.003 and 0.005, respectively). The area under the curve of the prediction models constructed by multivariate logistic regression analysis was 0.852. Figure 1 shows a comparison of the incidence rate of perioperative euDKA among the four groups: patients who discontinued and those who did not discontinue SGLT2is for ≥ 3 days prior to surgery and patients who received and those who did not receive insulin and glucose infusion. The highest incidence of perioperative euDKA was observed in patients who neither discontinued SGLT2is for ≥ 3 days nor received preoperative insulin and glucose infusion. Interestingly, among patients who did not discontinue SGLT2is, the incidence of perioperative euDKA was significantly lower in those who received preoperative insulin and glucose infusion than in those who did not receive preoperative insulin and glucose infusion (0.4% vs. 6.5%, *p* < 0.001; Fig. 1). No significant differences in all demographic characteristics were observed among the four groups (Supplementary Table 2). The median blood glucose levels within 30 days postoperatively also demonstrated no significant difference among the four groups (*p* = 0.079; Supplementary Table 3).

**Table 3:** Multivariate analysis of euDKA postoperatively up to 30 days

Variable	Odds ratio	95% CI	*p*-value
Age (years)	0.979	0.949–1.010	0.180
Male	1.202	0.467–3.091	0.703
BMI (kg/m^2^)	0.949	0.864–1.043	0.277
Discontinuation of SGLT2is for ≥ 3 days prior to surgery	0.047	0.006–0.353	0.003
Preoperative use of insulin and glucose infusion	0.054	0.007–0.408	0.005

BMI, body mass index; CI, confidence interval; SGLT2i, sodium-glucose cotransporter 2 inhibitor

## Discussion

In this present study, the multivariate analysis revealed the discontinuation of SGLT2is for ≥ 3 days prior to surgery and preoperative use of insulin and glucose infusion as significant factors influencing the incidence of perioperative euDKA. These findings suggest that preoperative SGLT2i discontinuation for at least 3 days could prevent perioperative euDKA development and that preoperative insulin and glucose infusion may further reduce the risk of developing euDKA, even in patients who cannot discontinue SGLT2is at least 3 days prior to surgery. To the best of our knowledge, this is the first cohort study to report the preventive effects of insulin and glucose infusion on perioperative euDKA development.

A previous study reported that the incidence of perioperative euDKA was 1.1% in patients taking SGLT2i and underwent emergency surgery [20], similar to that (1.8%) in the present study. In a case series describing episodes of SGLT2i-associated euDKA, surgery was a contributing factor in all patients with type 2 diabetes [9]. Moreover, another study of patients with diabetes receiving SGLT2is reported that the most common contributing factor for euDKA development was major surgery [19]. Lau et al. [13] reported that three patients on chronic empagliflozin therapy developed euDKA on postoperative day 1 following elective coronary artery bypass grafting surgery, despite discontinuing empagliflozin 24–48 h preoperatively. Considering the average 11–13 h half-life of SGLT2is, the last dose of SGLT2i may have to be administered no less than 55–65 h preoperatively for major elective surgical procedures [21]. A systematic review of case reports revealed that all 59 patients who developed perioperative euDKA did not discontinue SGLT2is ≥ 3 days prior to surgery [17]. In the present study, the incidence of perioperative euDKA in patients who did not discontinue SGLT2is ≥ 3 days prior to surgery without preoperative insulin and glucose infusion was 6.5% and was significantly higher than that in patients who discontinued SGLT2is ≥ 3 days prior to surgery without preoperative insulin and glucose infusion (0.4%, *p* < 0.001). These results imply that SGLT2i discontinuation for ≥ 3 days prior to surgery may be effective in preventing perioperative euDKA development.

In this study, among patients who did not discontinue SGLT2is at least 3 days prior to surgery, the incidence of perioperative euDKA in patients who received preoperative insulin and glucose infusion was significantly lower than that in patients who did not receive preoperative insulin and glucose infusion (0.4% vs. 6.5%, *p* < 0.001; Fig. 1). Recently, insulin and glucose infusion were implemented during emergency off-pump coronary artery bypass grafting in a patient with type 2 diabetes mellitus who received empagliflozin 2 days prior to surgery; the patient did not develop perioperative euDKA [22]. The glucose-lowering effect of SGLT2is is insulin independent, leading to a reduction in insulin secretion by pancreatic beta cells. Consequently, SGLT2i therapy results in insulin insufficiency. Lower blood insulin levels accelerate lipolysis and increase the production of free fatty acids, which are then converted to ketone bodies in the liver. SGLT2is also increase glucagon production. Lowering the insulin-to-glucagon ratio further stimulates lipolysis and increases free fatty acid and lipid oxygenation, resulting in ketoacidosis [1]. Therefore, we assume that insulin and glucose infusion play a role in covering the deficiency of insulin associated with SGLT2is therapy and that it could exert a preventive effect against perioperative euDKA in patients treated with SGLT2is. Furthermore, SGLT2is are approved for the treatment of heart failure as well as diabetics. Therefore, insulin and glucose infusion may be effective even in diabetic patients with heart failure who cannot discontinue SGLT2is at least 3 days preoperatively.

Perioperative euDKA did not develop in the majority of patients who did not discontinue SGLTis at least 3 days prior to surgery and receive insulin and glucose infusion. Nevertheless, the prevention of perioperative euDKA remains essential, given its potential as a life-threatening adverse event. Thus, this study proposing a new preventive strategy for perioperative euDKA would be useful to provide safe medical care.

To investigate the impact of emergency surgery on the development of perioperative euDKA, we compared the incidence of perioperative euDKA between patients who underwent emergency surgery (*n* = 327) and those who did not (*n* = 215). However, no significant difference was observed between the two groups. Thus, this result indicated that emergency surgery may exert minimal effect on the incidence of euDKA.

This study had certain limitations. First, the study could not confirm a causal relationship between SGLT2i use and perioperative ketoacidosis incidence, as perioperative euDKA may also be caused by various other factors, including the surgery itself and the use of other antidiabetic agents. Second, HbA1c level, which is reported to be a risk factor for perioperative euDKA [17], was recorded in only 582 patients (49.8%). Thus, we could not evaluate the impact of HbA1c levels on the development of euDKA and the logistic regression model cannot be adjusted for HbA1c. Third, this retrospective study involved patients from a single institution, and a potential selection bias regarding the preoperative use of insulin and glucose infusion could not be excluded. Furthermore, the incidence of euDKA in this study was low, as in a previous report, and it is possible that the influence of patient background factors associated with the incidence of perioperative euDKA other than insulin and glucose therapy and discontinuation of SGLT2is for ≥ 3 days prior to surgery was not fully analyzed. Future large-scale and multicenter prospective studies should be conducted to evaluate the safety and efficacy of insulin and glucose infusion and the factors affecting the development of perioperative euDKA. Finally, we did not determine the criteria for administration of insulin and glucose therapy and optimize the insulin-to-glucose ratio, which are necessary to establish the efficacy and safety of this preventive strategy for perioperative euDKA development, warranting further research.

## Conclusions

The findings of this study suggest that SGLT2i discontinuation for at least 3 days preoperatively can effectively prevent euDKA development. Insulin and glucose infusion prior to surgery will be beneficial in patients who cannot discontinue SGLT2is prior to an emergency surgery. This study provides novel evidence regarding the safety of the perioperative use of SGLT2is; the findings may guide the development of strategies for managing patients receiving SGLT2is pre-surgery.

## Supplementary Information

Below is the link to the electronic supplementary material.

Supplementary Material 1

Supplementary Material 2

Supplementary Material 3

## Data Availability

No datasets were generated or analysed during the current study.

## Abbreviations

ALT: Alanine aminotransferase
AST: Aspartate aminotransferase
BHBA: β-hydroxybutyrate
BMI: Body mass index
BUN: Blood urea nitrogen
CI: Confidence interval
euDKA: Euglycemic diabetic ketoacidosis
FDA: Food and Drug Administration
HbA1c: Glycated hemoglobin
N.A: Not available
OHA: Oral hypoglycemic agent
OR: Odds ratio
Scr: Serum creatinine
SGLT2i: Sodium-glucose cotransporter 2 inhibitor
