# Supplementary material for: Factors and preventive strategies for perioperative euglycemic diabetic ketoacidosis in patients with type 2 diabetes receiving sodium-glucose cotransporter 2 inhibitors: a retrospective study
Source: J Pharm Health Care Sci. 2025 Aug 25;11:79. doi: 10.1186/s40780-025-00487-6 (PMC12376744; doi:10.1186/s40780-025-00487-6)
Supplement: Supplementary file 1 — Supplementary Material 1 [file 40780_2025_487_MOESM1_ESM.pdf]

Supplementary Table 1. Perioperative outcomes

	Discontinuation of SGLT2is for ≥ 3 days prior to surgery		*p*-value
	(+) (n = 627)	(−) (n = 542)	
Surgical time (min)	186 [29–753]	183 [23–759]	0.352
Surgical blood loss (mL)	216 [0–17,255]	200 [0–16,880]	0.646
Type of surgery			
Coronary artery bypass Grafting	228 (36.4)	195 (36.0)	0.998
Gastrointestinal resection	167 (26.6)	153 (28.2)	
Cerebral aneurysm clopping	54 (8.6)	43 (7.9)	
Brain tumor resection	38 (6.1)	34 (6.3)	
Laminectomy	33 (5.3)	26 (4.8)	
Lumber spine fusion	31 (4.9)	23 (4.2)	
Total knee replacement	26 (4.1)	26 (4.8)	
Bariatric surgery	25 (4.0)	20 (3.7)	
Cerebral revascularization	18 (2.9)	17 (3.1)	
Others	7 (1.1)	5 (0.9)	
Intraoperative laboratory parameter			
Blood glucose level (mg/dL)	110 [65–236]	109 [69–233]	0.357

Values are presented as median [range] or number (%). The data were analyzed using the Mann–Whitney U test or Fisher's exact test. SGLT2i, sodium-glucose cotransporter 2 inhibitor.
