# Supplementary material for: Factors and preventive strategies for perioperative euglycemic diabetic ketoacidosis in patients with type 2 diabetes receiving sodium-glucose cotransporter 2 inhibitors: a retrospective study
Source: J Pharm Health Care Sci. 2025 Aug 25;11:79. doi: 10.1186/s40780-025-00487-6 (PMC12376744; doi:10.1186/s40780-025-00487-6)
Supplement: Supplementary file 2 — Supplementary Material 2 [file 40780_2025_487_MOESM2_ESM.pdf]

Supplementary Table 2. Characteristics of the patients

Discontinuation of SGLT2is for ≥ 3 days prior to surgery	(+)		(−)		*p*-value
Perioperative use of insulin and glucose infusion	(+) (n = 342)	(−) (n = 285)	(+) (n = 250)	(−) (n = 292)	
Age (years)	70 [26–90]	67 [31–89]	65 [35–97]	66 [27–96]	0.825
Male	223 (65.2)	174 (61.1)	155 (62.0)	182 (62.3)	0.726
BMI (kg/m^2)	24.2 [13.6–38.7]	24.3 [13.1–39.2]	23.8 [15.4–39.6]	23.9 [14.4–38.3]	0.498
SGLT2i type					
Dapagliflozin	99 (28.9)	83 (29.1)	73 (29.2)	91 (31.2)	0.638
Empagliflozin	92 (26.9)	79 (27.7)	64 (25.6)	89 (30.5)	
Canagliflozin	69 (20.2)	49 (17.2)	40 (16.0)	50 (17.1)	
Ipragliflozin	37 (10.8)	36 (12.6)	40 (16.0)	33 (11.3)	
Tofogliflozin	28 (8.2)	18 (6.3)	22 (8.8)	19 (6.5)	
Luseogliflozin	17 (5.0)	20 (7.0)	11 (4.4)	10 (3.4)	
Other anti-diabetic medications					
None	305 (89.2)	252 (88.4)	222 (88.8)	260 (89.0)	0.992
OHA	31 (9.1)	28 (9.8)	24 (9.6)	27 (9.2)	0.989
Insulin	5 (1.5)	5 (1.8)	4 (1.6)	5 (1.7)	0.992
Insulin and OHA	1 (0.3)	0 (0.0)	0 (0.0)	0 (0.0)	0.490
Baseline laboratory parameters					
AST level (U/L)	21 [10–73]	21 [8–74]	20 [7–74]	21 [8–74]	0.252
ALT level (U/L)	16 [3–74]	16 [3–74]	16 [5–74]	16 [3–70]	0.501
Scr level (mg/dL)	0.84 [0.40–6.88]	0.83 [0.28–6.87]	0.80 [0.36–6.76]	0.83 [0.36–6.89]	0.194
BUN level (mg/dL)	17 [7–91]	17 [5–88]	18 [7–78]	18 [6–82]	0.340
Serum sodium level (mEq/L)	140 [121–148]	140 [118–152]	140 [123–151]	139 [120–148]	0.227
Serum potassium level (mEq/L)	4.2 [3.1–5.7]	4.1 [3.0–6.0]	4.2 [3.2–6.0]	4.2 [3.2–6.0]	0.118
Serum chloride level (mEq/L)	106 [97–118]	105 [92–119]	105 [89–118]	105 [94–114]	0.112
Arterial pH	7.45 [7.38–7.48]	7.43 [7.34–7.83]	7.44 [7.43–7.56]	7.43 [7.33–7.67]	0.779
Serum bicarbonate level (mEq/L)	23.9 [18.1–30.8]	24.0 [17.0–31.8]	24.3 [15.9–31.5]	24.1 [18.2–30.0]	0.544
Serum BHBA level (μmol/L)	38 [16–174]	39 [15–178]	35 [14–172]	36 [14–174]	0.988
Blood glucose level (mg/dL)	124 [69–295]	120 [66–281]	125 [71–292]	119 [63–290]	0.334

Values are presented as median [range] or number (%). The data were analyzed using the Kruskal–Wallis test or a Chi-square test of independence. ALT, alanine transaminase; AST, aspartate transaminase; BHBA, β-hydroxybutyrate; BMI, body mass index; BUN, blood urea nitrogen; OHA, oral hypoglycemic agent; Scr, serum creatinine; SGLT2i, sodium-glucose cotransporter 2 inhibitor.
