# Supplementary material for: Factors and preventive strategies for perioperative euglycemic diabetic ketoacidosis in patients with type 2 diabetes receiving sodium-glucose cotransporter 2 inhibitors: a retrospective study
Source: J Pharm Health Care Sci. 2025 Aug 25;11:79. doi: 10.1186/s40780-025-00487-6 (PMC12376744; doi:10.1186/s40780-025-00487-6)
Supplement: Supplementary file 3 — Supplementary Material 3 [file 40780_2025_487_MOESM3_ESM.pdf]

Supplementary Table 3. Perioperative and postoperative outcomes

Discontinuation of SGLT2is for ≥ 3 days prior to surgery	(+)		(−)		*p*-value
Perioperative use of insulin and glucose infusion	(+) (n = 342)	(−) (n = 285)	(+) (n = 250)	(−) (n = 292)	
Surgical time (min)	196 [29–753]	181 [30–697]	187 [23–759]	178 [29–622]	0.111
Surgical blood loss (mL)	210 [0–17,255]	222 [0–15,140]	205 [0–15,020]	195 [0–16,880]	0.102
Type of surgery					
Coronary artery bypass grafting	134 (39.2)	94 (33.0)	82 (32.8)	113 (38.7)	0.997
Gastrointestinal resection	92 (26.9)	75 (26.3)	75 (30.0)	78 (26.7)	
Cerebral aneurysm clopping	29 (8.5)	25 (8.8)	22 (8.8)	21 (7.2)	
Brain tumor resection	20 (5.8)	18 (6.3)	17 (6.8)	17 (5.8)	
Laminectomy	17 (5.0)	16 (5.6)	12 (4.8)	14 (4.8)	
Lumber spine fusion	15 (4.4)	16 (5.6)	11 (4.4)	12 (4.1)	
Total knee replacement	12 (3.5)	14 (4.9)	13 (5.2)	13 (4.5)	
Bariatric surgery	11 (3.2)	14 (4.9)	9 (3.6)	11 (3.8)	
Cerebral revascularization	7 (2.0)	11 (3.9)	7 (2.8)	10 (3.4)	
Others	5 (1.5)	2 (0.7)	2 (0.8)	3 (1.0)	
Intraoperative laboratory parameter					
Blood glucose level (mg/dL)	111 [70–180]	109 [65–236]	108 [69–215]	112 [71–233]	0.116
Predominant symptoms of euDKA					
Nausea	59 (17.3)	50 (17.5)	45 (18.0)	68 (23.3)	0.193
Vomiting	21 (6.1)	19 (6.7)	19 (7.6)	19 (6.5)	0.916
Tachycardia	0 (0.0)	1 (0.4)	1 (0.4)	4 (1.4)	0.104
Abdominal pain	0 (0.0)	0 (0.0)	1 (0.4)	1 (0.3)	0.504
Postoperative laboratory parameters					
Arterial pH	7.48 [7.39–7.49]	7.42 [7.32–7.76]	7.45 [7.44–7.55]	7.35 [7.27–7.55]	0.009
Serum bicarbonate level (mEq/L)	25.1 [20.0–36.6]	24.9 [17.0–32.5]	25.0 [19.8–33.1]	23.3 [11.7–28.9]	0.002
Serum BHBA level (μmol/L)	33 [11–185]	38 [12–203]	32 [12–201]	41 [22–574]	0.117
Blood glucose level (mg/dL)	114 [75–232]	112 [68–257]	109 [70–234]	114 [74–282]	0.079
Level of postoperative blood glucose					
Hyperglycemia (≥ 125 mg/dL)	85 (24.9)	82 (28.8)	61 (24.4)	86 (29.5)	0.392
Mild hyperglycemia (125–250 mg/dL)	81 (95.3)	77 (93.9)	57 (93.4)	78 (90.7)	0.566
Hypoglycemia (≤ 60 mg/dL)	0 (0.0)	0 (0.0)	0 (0.0)	0 (0.0)	N.A.
Days until eating and SGLT2i are resumed after surgery	1 [0–23]	1 [0–15]	1 [0–24]	1 [0–18]	0.154

Values are presented as median [range] or number (%). The data were analyzed using the Kruskal–Wallis test or a Chi-square test of independence. BHBA, β-hydroxybutyrate; euDKA, euglycemic diabetic ketoacidosis; N.A., not available; SGLT2i, sodium-glucose

cotransporter 2 inhibitor.
